# Patterns of Clinical Response with Talimogene Laherparepvec (T-VEC) in Patients with Melanoma Treated in the OPTiM Phase III Clinical Trial

**DOI:** 10.1245/s10434-016-5286-0

**Published:** 2016-06-24

**Authors:** Robert H. I. Andtbacka, Merrick Ross, Igor Puzanov, Mohammed Milhem, Frances Collichio, Keith A. Delman, Thomas Amatruda, Jonathan S. Zager, Lee Cranmer, Eddy Hsueh, Lisa Chen, Mark Shilkrut, Howard L. Kaufman

**Affiliations:** 1 Huntsman Cancer Institute, University of Utah, Salt Lake City, UT USA; 2 University of Texas MD Anderson Cancer Center, Houston, TX USA; 3 Vanderbilt University Medical Center, Nashville, TN USA; 4 University of Iowa Hospitals and Clinics, Iowa City, IA USA; 5 University of North Carolina, Chapel Hill, NC USA; 6 Emory University, Atlanta, GA USA; 7 Minnesota Oncology, Fridley, MN USA; 8 Moffitt Cancer Center, Tampa, FL USA; 9 University of Washington School of Medicine, Seattle, WA USA; 10 Saint Louis University Cancer Center, St Louis, MO USA; 11 Amgen Inc., Thousand Oaks, CA USA; 12 Rutgers Cancer Institute of New Jersey, Rutgers, NJ USA

## Abstract

**Purpose:**

Talimogene laherparepvec (T-VEC) is an oncolytic immunotherapy designed to induce tumor regression of injected lesions through direct lytic effects, and of uninjected lesions through induction of systemic antitumor immunity. In this study, we describe the patterns and time course of response to T-VEC from the phase III OPTiM trial of 436 patients with unresected stages IIIB–IV melanoma.

**Methods:**

Lesion-level response analyses were performed based on the type of lesion (injected or uninjected cutaneous, subcutaneous, or nodal lesions; or visceral lesions [uninjected]), and the best percentage change from baseline of the sum of products of the longest diameters was calculated. Patients randomized to T-VEC (*n* = 295) who experienced a durable response (continuous partial or complete response for ≥6 months) were evaluated for progression prior to response (PPR), defined as the appearance of a new lesion or >25 % increase in total baseline tumor area.

**Results:**

T-VEC resulted in a decrease in size by ≥50 % in 64 % of injected lesions (*N* = 2116), 34 % of uninjected non-visceral lesions (*N* = 981), and 15 % of visceral lesions (*N* = 177). Complete resolution of lesions occurred in 47 % of injected lesions, 22 % of uninjected non-visceral lesions, and 9 % of visceral lesions. Of 48 patients with durable responses, 23 (48 %) experienced PPR, including 14 who developed new lesions only. No difference in overall survival was observed, and median duration of response was not reached in patients with PPR versus those without PPR.

**Conclusions:**

Responses in uninjected lesions provide validation of T-VEC-induced systemic immunotherapeutic effects against melanoma. PPR did not negatively impact the clinical effectiveness of T-VEC.

**Electronic supplementary material:**

The online version of this article (doi:10.1245/s10434-016-5286-0) contains supplementary material, which is available to authorized users.

Immunotherapies have proven to be powerful and effective treatments for patients with advanced melanoma. Talimogene laherparepvec (T-VEC) is a herpes simplex virus (HSV) type 1-based oncolytic immunotherapy designed to replicate selectively in tumors and produce human granulocyte–macrophage colony-stimulating factor (GM-CSF).^1^,^2^ In the randomized phase III OPTiM trial of patients with unresectable stage IIIB–IV melanoma, intralesional T-VEC administration yielded an improvement in the primary endpoint of durable response rate (DRR; defined as a response beginning in the first 12 months of treatment and lasting at least 6 months continuously) versus subcutaneous administration of GM-CSF (16 vs. 2 %; odds ratio 8.9; *p* < 0.0001).^3^ Overall response rate (ORR) was 26 % [11 % complete response (CR)] in patients treated with T-VEC, and 6 % (1 % CR) in patients treated with GM-CSF. Median overall survival (OS; a secondary endpoint) in the T-VEC arm was 23.3 versus 18.9 months in the GM-CSF arm (hazard ratio [HR] 0.79, 95 % confidence interval [CI] 0.62–1.00; *p* = 0.051).^3^

T-VEC may induce tumor regression both through direct lytic effects following intratumoral injection into tumors and through secondary induction of systemic antitumoral immunity in the context of virally mediated GM-CSF production.^1^,^4^ The direct lytic effects are expected to mediate rapid tumor responses in injected lesions. Induction of systemic immunity may require more time to prime antigen-specific T cell responses, but could lead to regression of uninjected tumors harboring shared tumor-derived antigens with injected lesions. Similar dynamics of antitumor response have been proposed for other immunotherapeutic agents, such as with PD-1 pathway inhibitors^5^–^7^ and anti-CTLA-4 inhibitors,^6^,^7^ with some patients experiencing tumor progression prior to eventual regression. Thus, the onset of immune-mediated tumor responses may be delayed compared with the immediate effects of cytotoxic agents and tyrosine kinase inhibitors.

Since responses to T-VEC in uninjected sites were documented in some patients with melanoma in phase I and II studies,^2^,^4^ we sought to validate, quantify, and characterize the systemic effects of T-VEC in patients in OPTiM. We compared response patterns of injected and uninjected tumors in T-VEC-treated patients and conducted an analysis of overall responses to assess whether T-VEC, as with other immunotherapies, induces delayed antitumor responses.

## Materials and Methods

### OPTiM Trial Design and Treatment

In this open-label, multicenter, phase III study, 436 patients with previously treated and untreated, unresected, stage IIIB–IV melanoma were randomized 2:1 to receive intralesional T-VEC or subcutaneous GM-CSF. The primary endpoint of OPTiM was DRR; key secondary endpoints included ORR, OS, and safety. The clinical trial design, treatment, and primary results have been reported.^3^ All participating sites had approval from the Institutional Review Boards or Ethics Committees, and all participants provided written, informed consent.

A treatment cycle of T-VEC consisted of two consecutive injections (5 weeks for the first cycle and 4 weeks for subsequent cycles). At each treatment session, injecting new lesions followed by larger lesions was prioritized. Visceral lesions were not allowed to be injected. Treatment continued for at least 6 months, during which treatment discontinuation for disease progression was not required; an increase in lesion size or appearance of new lesions was expected to occur in some patients based on the results from a phase II study.^2^ After 6 months, treatment continued until clinically significant disease progression was documented in association with a decline in performance status, intolerability, or lack of injectable lesions.

### Assessments

Visible or palpable lesions were evaluated by clinical evaluation (caliper or ruler) at baseline and day 1 of each treatment cycle. Deeper subcutaneous, nodal, or visceral lesions were assessed by computed tomography (CT), positron emission tomography/CT, and ultrasonography, if appropriate, and performed at baseline and every 12 weeks. Overall tumor response was determined by WHO criteria^8^ modified to allow patients who developed new lesions or increase in lesion size to be evaluated for tumor response later.^3^ In the event of response, any residual cutaneous pigmented areas or other residual masses had to be documented as not containing tumor by a representative biopsy. In addition, investigators were encouraged to take biopsies of residual pigmented areas or masses suspected of no longer containing the tumor at any time point during the study. A blinded independent Endpoint Assessment Committee (EAC) evaluated patients with a best response per investigator of CR or PR, or who received treatment for ≥9 months, by reviewing photographs of all visible lesions, other imaging assessments, and biopsy results. Investigator measurements of individual lesions and assessments of response were also collected and analyzed for this report.

### Lesion-Level Response Analysis

Since EAC-derived measurements were only available for responders per investigator or those who had at a ≥9-month treatment period, investigator-reported assessments in 291 patients treated with T-VEC (four patients randomized to T-VEC did not receive treatment) were used to evaluate response of individual lesions during treatment. The site, frequency, and location of injections were recorded at the beginning of each treatment cycle.

For this analysis, lesions were considered evaluable for response if two or more measurements were recorded at two separate time points. Evaluations of individual lesion responses were conducted using the best percentage change from baseline with a cutoff of ≥50 % decrease in tumor lesion size. This was defined as a product of the two largest perpendicular diameters. Lesion-level response analyses were calculated based on the type of lesion: injected (recorded as having been injected), uninjected non-visceral (non-visceral lesions never recorded as having been injected), and visceral (identified by medical review of investigator-described locations of sites of the disease; per protocol, injection of these lesions was not permitted). Locations of visible or palpable tumor lesions were also assigned to a body-site grid.

### Progression Prior to Response Analysis

Patients randomized to the T-VEC arm who experienced a DR were evaluated for PPR, defined as the appearance of a new lesion or >25 % increase in baseline total tumor area (the sum of the products of the two largest perpendicular diameters of all index lesions at baseline). Since all data for responders per investigators were also assessed by an independent, treatment-blinded EAC, EAC-reported measurements were used for the PPR analysis. Responders by EAC were grouped as with or without PPR. Responders with PPR were further subdivided by progression in existing lesions (with or without new lesions) or with appearance of new lesions only. Responders without PPR were further divided by durable response (DR) onset at ≤6 months from receiving initial T-VEC treatment, or DR onset after 6 months from receiving initial T-VEC treatment.

## Results

Demographics and disease characteristics of T-VEC treated patients in OPTiM are shown in electronic supplementary Appendix 1. Among 295 patients randomized to the T-VEC arm, four patients did not receive the allocated treatment. The median number of lesions per T-VEC-treated patient was 10 (range 1–58; Electronic Supplementary Appendix 2), median number of lesions injected with T-VEC per patient over the duration of the study was 5 (range 1–56; Electronic Supplementary Appendix 2), and the mean volume of T-VEC administered per treatment was 2.8 mL (interquartile range [IQR] 1.8–4.0).

### Response of lesions to T-VEC

To separate local and systemic antitumor effects associated with T-VEC treatment, analysis of changes in tumor size as reported by investigators was conducted on 3274 evaluable baseline and new lesions from 285 evaluable patients (6 of 291 patients who received T-VEC treatment did not have two or more measurements for evaluating tumor area change). Overall, 259 (91 %) of the 285 evaluable patients had three or more lesions.

### Injected Lesions

**Fig. 1:**
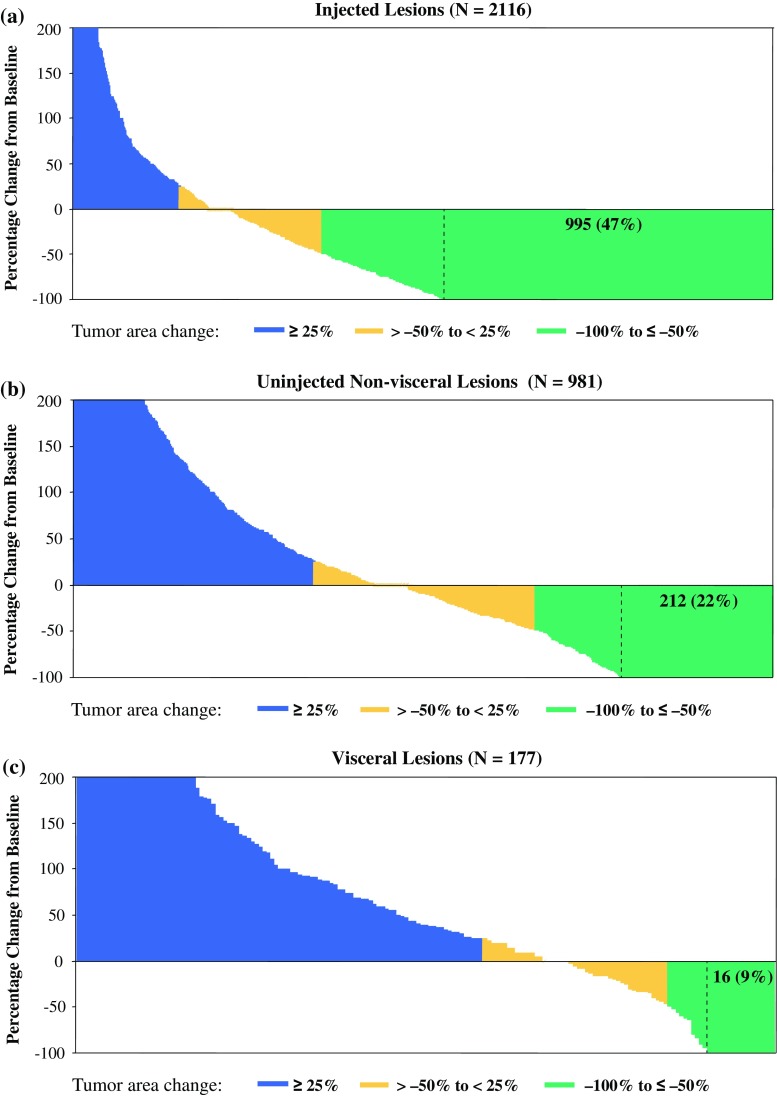
T-VEC administration generated response in both injected and uninjected tumor lesions, including visceral lesions. Response of **a** individual injected lesions; **b** uninjected non-visceral lesions; and **c** visceral lesions (also uninjected). Vertical axis depicts maximal change in individual tumor lesion size (products of the two largest perpendicular diameters) from baseline

A total of 2116 individual lesions injected with T-VEC from 277 patients were evaluable per investigator assessment. Of these, 1361 (64 %) lesions had a decrease in size of ≥50 % (best percentage change from baseline), including 995 (47 %) lesions that resolved completely (Fig. 1a). Median time to response of responding injected lesions from baseline was 9.3 weeks (IQR 5.1–17.1 weeks; Electronic Supplementary Appendix 3). We next assessed the relationship between regression in individual injected lesions and overall patient responses (by modified WHO criteria). Among 277 patients evaluable for response in injected lesions, 37 % had ≥50 % decrease in total tumor area of injected lesions and 16 % had complete resolution of injected lesions. The ORR by investigator in these patients was 32 %, with 15 % having a CR (Electronic Supplementary Appendix 4).

### Uninjected Non-visceral Lesions

Overall, 981 individual, uninjected, non-visceral lesions (cutaneous, subcutaneous, or nodal) from 177 patients were evaluable per investigator assessment. Of these, 331 (34 %) uninjected, non-visceral lesions decreased in size by ≥50 %, and 212 (22 %) resolved completely (Fig. 1b). Of 331 lesions that decreased in size by ≥50 %, 159 (48 %) were located in the same body site as injected lesions and 77 (23 %) were located in a different body site [95 (29 %) were not assigned body-site area; see Electronic Supplementary Appendix 5 for additional details]. Median time to response of responding uninjected non-visceral lesions from baseline was 12.9 weeks (IQR 9.1–20.9 weeks; Electronic Supplementary Appendix 3). Among 177 patients evaluable for response in uninjected non-visceral lesions, 21 % had ≥50 % decrease in total tumor area of uninjected non-visceral lesions and 14 % had complete resolution of uninjected non-visceral lesions, which corresponded to an ORR of 18 % and a CR rate of 6 % (Electronic Supplementary Appendix 6).

### Visceral Lesions

Overall, 177 individual visceral lesions from 79 patients were evaluable per investigator assessment. Twenty-seven (15 %) visceral lesions decreased in size by ≥50 %, and 16 (9 %) resolved completely (Fig. 1c). Evaluable visceral lesions were located mainly in the lung (77 %), and also in the liver (8 %), adrenal gland (6 %), spleen (3 %), kidney (3 %), pancreas (1 %), and the thyroid, brain, and gastrointestinal tract (<1 % each). Among responding visceral lesions, 81 % were in the lung, 15 % in the liver, and 4 % in the thyroid. Median time to response of responding visceral lesions from baseline was 12.3 weeks (IQR 11.4–36.9; Electronic Supplementary Appendix 3). Among 79 patients evaluable for response in visceral lesions, 10 % had a ≥50 % decrease in total tumor area of visceral lesions and 6 % had complete resolution of visceral lesions. ORR was 14 % and the CR rate was 3 % (Electronic Supplementary Appendix 7).

## Patterns of Response in T-VEC-Treated Patients

**Fig. 2:**
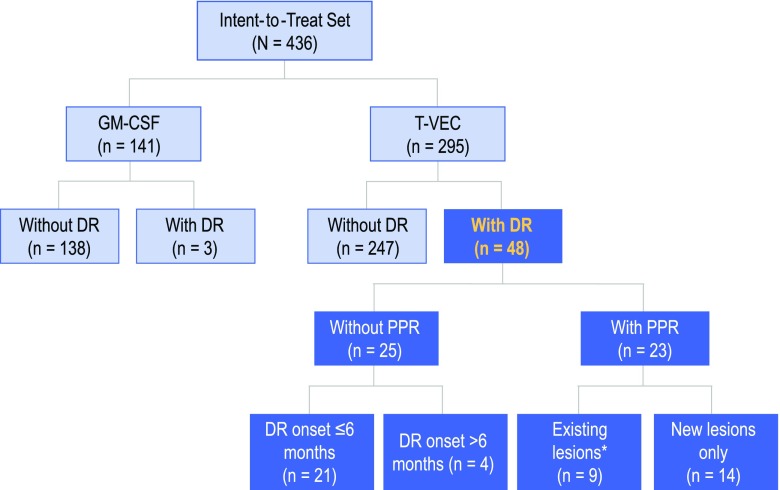
Analysis of the patterns of response in T-VEC-treated patients in OPTiM. * Includes three patients with PPR in existing lesions who may have also developed new lesions. *PPR* progression prior to response, *GM*-*CSF* granulocyte–macrophage colony-stimulating factor, *DR* durable response

In OPTiM, 295 were randomized to T-VEC and 141 to GM-CSF (Fig. 2). In the GM-CSF arm, only three (2 %) patients experienced a DR as per the EAC, therefore the current analysis was limited to the T-VEC arm. Forty-eight patients (16 %) treated with T-VEC experienced a DR (Fig. 2). Of these, 25 (52 %) did not have PPR and 23 (48 %) had PPR. Forty (83 %) of 48 T-VEC-treated patients with a DR had responses ongoing at the time of this analysis (range 10.8–19.2 months; median follow-up 18.4 months). Of these 40 patients with ongoing DR, 18 did not have PPR and 22 had PPR (*p* = 0.27).

In 48 T-VEC-treated patients with a DR, median time to DR onset was 3.1 months (range 1.2–9.5 months) in patients without PPR versus 5.8 months (range1.3–10.6 months) in patients with a PPR (*p* = 0.004). Median duration of DR was not reached in both groups, with a minimum duration of response of 6.3 months for patients with PPR and 6.2 months for patients without PRR (the response with shortest duration was ongoing at time of this analysis). In unadjusted comparison of durable responders with PPR and those without PRR, no difference in OS was observed (log rank *p* = 0.35, HR 0.35, 95 % CI 0.04–3.44; Electronic Supplementary Appendix 8). Median OS was not reached for either group at the time of this analysis.

**Fig. 3:**
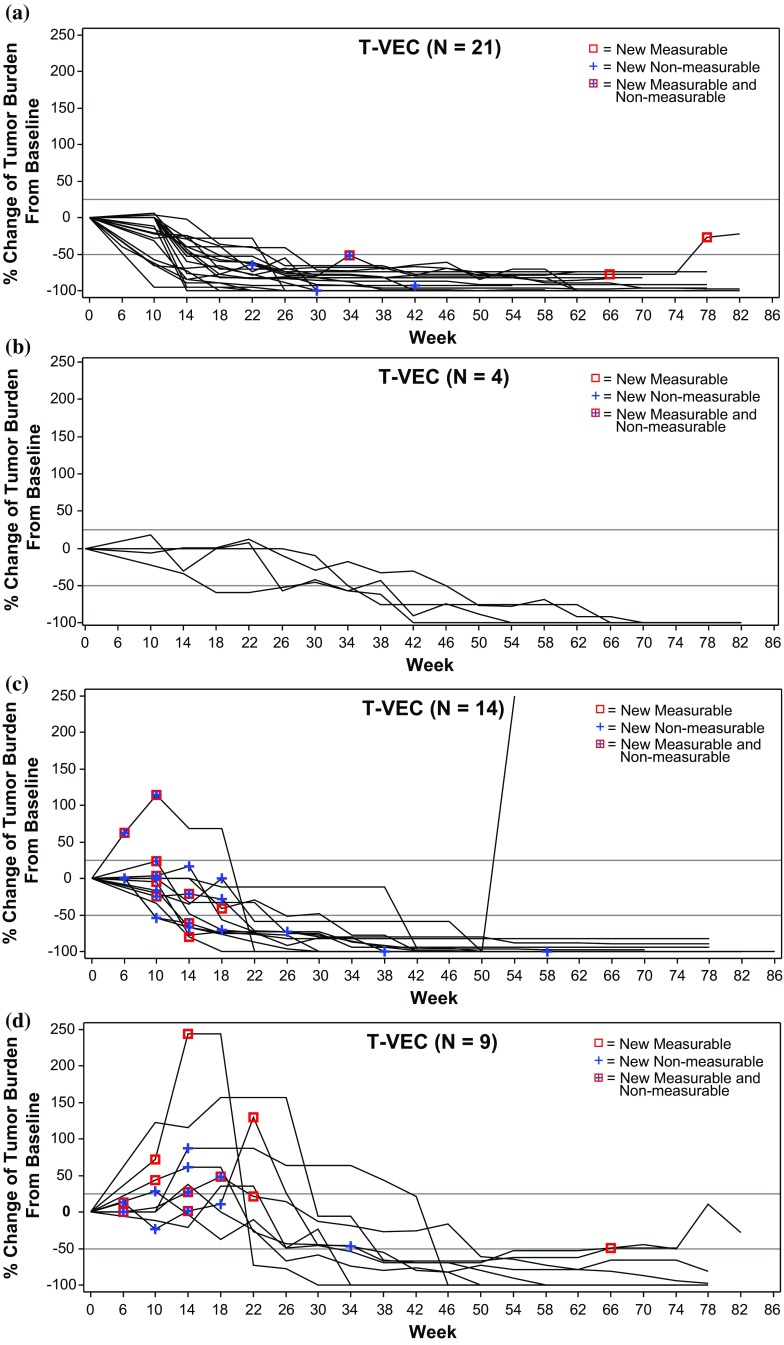
Four distinct patterns of response in T-VEC-treated patients. **a** Without PPR and DR onset ≤6 months; **b** without PPR and DR onset >6 months; **c** with PPR due to new lesions only; and **d** with PPR due to existing lesions (with or without new lesions). The vertical axis depicts the change in tumor area from baseline, as assessed by the Endpoint Assessment Committee. *PPR* progression prior to response, *DR* durable response

**Fig. 4:**
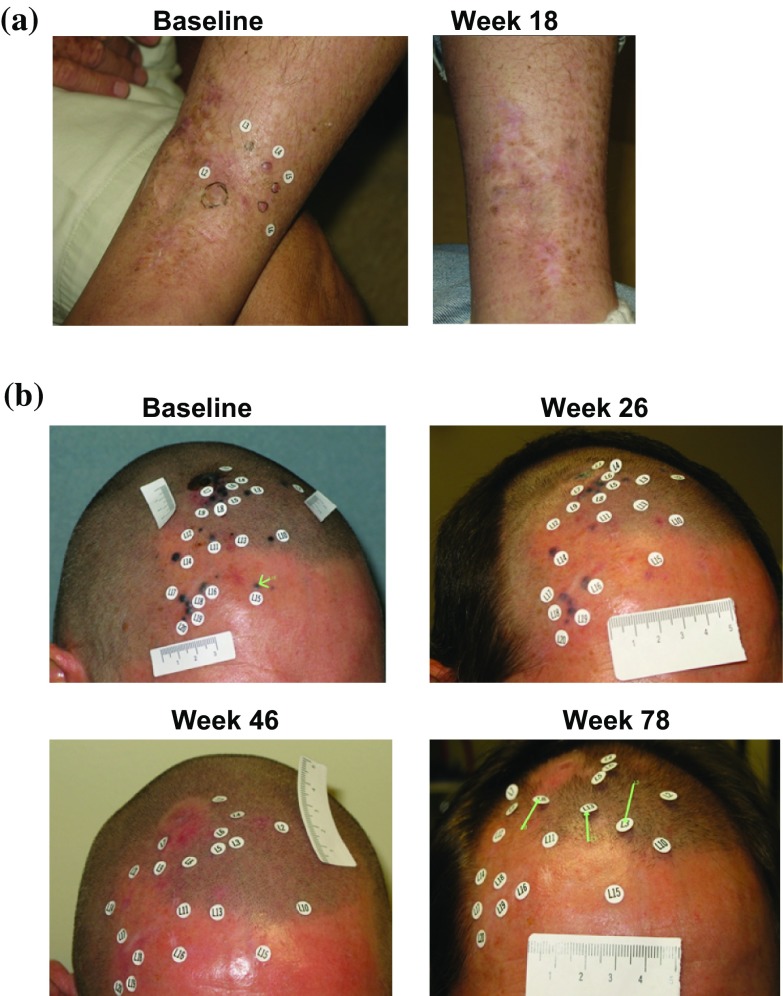
Examples of patients treated with T-VEC with a DR without PPR. **a** Patient without PPR and DR onset ≤6 months. The patient had recurrent stage IIIC melanoma with multiple in-transit tumor lesions on the leg. All lesions were injected with T-VEC and all resolved (CR) by 37 weeks after the start of treatment. The patient remained in CR until the end of the study, with DR duration of 60 weeks. **b** Patient without PPR and measurable response onset >6 months. The patient had recurrent stage IIIB (in-transit) melanoma of the scalp with 20 cutaneous lesions that were injected with T-VEC. Partial response was recorded on week 30 after the start of treatment. Lesions resolved completely by week 38 and the patient remained in CR until the end of the study, with DR duration of 48 weeks. Responses are reported per External Assessment Committee. Titles above each photography are weeks on study. *DR* durable response, *PRR* progression prior to response, *CR* complete response

Of 25 patients without PPR, 21 (84 %) had a DR that began within the first 6 months of receiving T-VEC (Fig. 2). Figure 3a depicts changes in tumor area from baseline of these patients, and examples are shown in Fig. 4a and Electronic Supplementary Appendix 9. Four (16 %) patients without PPR had a DR that began after 6 months (Fig. 3b; see Fig. 4b for an example).

**Fig. 5:**
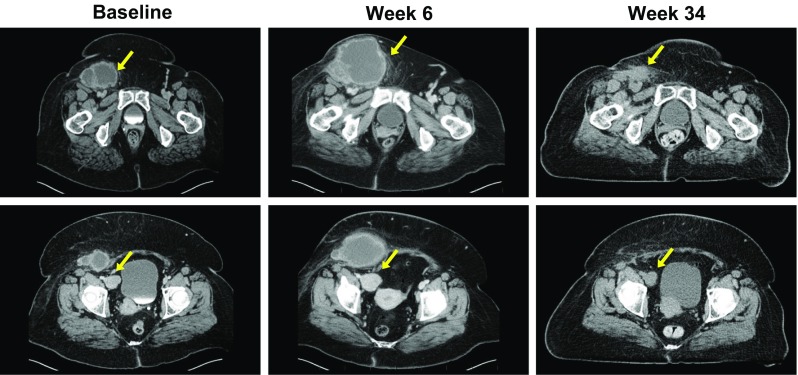
Example of a patient treated with T-VEC with a durable response and PPR due to enlargement of existing lesions. The patient had recurrent stage IVM1c melanoma of the right lower extremity with multiple regional and distant nodal metastases and elevated serum lactic dehydrogenase. Right inguinal and iliac lymph nodes were injected with T-VEC under ultrasound guidance (from week 14 for the iliac lymph node). PPR and increase in size in both injected and uninjected lesions were documented during the first 26 weeks of treatment. Responses are reported per External Assessment Committee. Titles above each photography are weeks on study. Partial response was recorded on week 40, and continued for 28 weeks. *PPR* progression prior to response

In 23 patients with PPR, 14 (61 %) experienced progression due to the appearance of a new lesion(s) prior to DR onset (Fig. 3c; see Electronic Supplementary Appendix 10 for an example) and 9 (39 %) experienced PPR due to an increase in the size of existing lesions with or without the appearance of new lesion(s) (Fig. 3d; see Fig. 5 for an example).

## Discussion

In this study, T-VEC administration yielded regression of ≥50 % in 64 % of injected lesions (with approximately half of the injected lesions resolving completely) and in uninjected lesions (34 % in non-visceral and 15 % in visceral). Delayed tumor responses due to PPR occurred in approximately half of the T-VEC-treated patients with DRs. PPR did not appear to significantly impact the duration and quality of DRs or OS.

Local tumor lysis achieved by direct injection of T-VEC into the lesion is believed to lead to release of tumor-derived antigens, and virally encoded GM-CSF potentiates systemic antitumor immune responses. Preclinical results indicated that incorporation of GM-CSF into the oncolytic virus was important for the systemic antitumor effect of T-VEC since this resulted in increased tumor shrinkage of uninjected tumors in mice.^1^ In a phase II study in patients with melanoma,^9^ an ORR of 26 % was reported, with responses frequently observed in uninjected lesions, including visceral lesions. Supporting evidence of the systemic effect of T-VEC came from biopsies that were taken from injected and uninjected lesions in 11 of the 50 patients treated with T-VEC. Analysis of regressing lesions, including uninjected lesions, suggested an association between response and the presence of MART-1-specific CD8^+^ T cells and reduction in CD4^+^ regulatory T cells, consistent with induction of host antitumor immunity.^9^

To further understand the systemic effects of T-VEC, we performed an analysis of the response of individual lesions from patients in the phase III OPTiM trial. Consistent with previous observations,^9^ T-VEC administration resulted in responses in both injected and uninjected lesions. These individual lesion responses occurred earlier in injected lesions, likely due to the time required for a systemic immune response to be established. The systemic effects of T-VEC were unlikely due to direct exposure of uninjected lesions to the oncolytic virus from adjacent injected lesions, as responses were seen in visceral lesions and in uninjected non-visceral lesions located in different body sites than injected lesions.

Similar to other immunotherapies,^10^ some T-VEC-treated patients experienced PPR. Nearly half of all T-VEC durable responders experienced PPR, with the majority of these progression events being due to development of new lesions. While DR occurred nearly 3 months earlier in patients without PPR versus those with PPR, the percentage of patients with a DR ongoing at the time of this analysis was similar in both groups. Thus, PPR did not seem to adversely impact the duration of response.

Responses after an initial increase in tumor size or appearance of new lesions have been observed in patients with melanoma treated with immunotherapies such as ipilimumab (10 % of treated patients who were initially characterized with disease progression),^10^,^11^ nivolumab (4 % of all treated patients),^5^,^6^ and pembrolizumab (9 % of evaluable treated patients).^7^ In addition to melanoma, PPR (pseudoprogression) was reported for other tumor types treated with checkpoint inhibitors.^12^ Therefore, the occurrence of subsequent responses, including complete disease resolution that occurs in some patients, may justify continued treatment with immunotherapies despite early disease progression. Distinguishing true progression from pseudoprogression is likely to be a challenge with T-VEC and other immunotherapies that display delayed tumor responses due to inflammatory tumor infiltrates.^13^,^14^

In this study, patients with PPR relative to those without PPR had younger median age, better Eastern Cooperative Oncology Group (ECOG) performance status, more HSV-1 seronegativity at baseline, more female patients, and more patients who received T-VEC as ≥2 line of therapy. However, due to the small numbers, as well as unknown data for potential confounding characteristics (e.g. *BRAF* status), formal comparisons were impractical. Research in a larger cohort of patients as well as parallel efforts to find biomarkers are needed to help identify patients who may experience true progression versus pseudoprogression.

Conventional response criteria such as RECIST^15^ and WHO^8^ that rely on tumor shrinkage subsequent to treatment as an indicator of antitumor activity may underestimate the full benefit of immunotherapies.^12^ Immune-related response criteria, in which new lesions are incorporated into the total tumor area and disease progression criteria, and require confirmation of ≥25 % increase in total tumor area ≥4 weeks later, have been proposed to address distinct response patterns observed with immunotherapies, specifically disease progression followed by response.^10^ The data reported here and by others^5^,^6^,^10^,^11^ provide additional support for the use of revised response criteria in evaluating immunotherapies.

## Conclusions

T-VEC demonstrates response in injected and uninjected lesions, and represents a new first-in-class oncolytic virus immunotherapy for patients with melanoma. These data also suggest that, similar to other novel immunotherapies, more patients may have benefited from treatment with T-VEC due to the delayed nature of tumor regression. Opportunities to enhance T-VEC activity could include direct injection of visceral lesions and/or combination with immune checkpoint inhibitors. Early reports from a phase Ib clinical trial of T-VEC and ipilimumab suggest a response rate of 56 % and a CR rate of 33 %; a randomized phase II study is ongoing.^16^ Future studies will likely focus on defining the specific mechanisms by which T-VEC mediates tumor regression and the development of rational combination therapies to improve the potential of this agent for patients with cancer, in both melanoma and other tumors.

## Electronic supplementary material

Below is the link to the electronic supplementary material.

Supplementary material 1 (DOCX 1260 kb)
