# Supplementary material for: Patterns of Clinical Response with Talimogene Laherparepvec (T-VEC) in Patients with Melanoma Treated in the OPTiM Phase III Clinical Trial
Source: Ann Surg Oncol. 2016 Jun 24;23(13):4169–77. doi: 10.1245/s10434-016-5286-0 (PMC5090012; doi:10.1245/s10434-016-5286-0)

**APPENDICES**

Patterns of clinical response with talimogene laherparepvec (T-VEC) in patients with melanoma treated in the OPTiM Phase III clinical trial.

Robert H.I. Andtbacka, MD, CM, FACS FRCSC,^1^ Merrick Ross, MD,^2^  Igor Puzanov, MD, MSI, FACP,^3^ Mohammed Milhem. MD,^4^ Frances Collichio, MD,^5^ Keith A. Delman, MD, FACS,^6^ Thomas Amatruda, MD,^7^ Jonathan S. Zager, MD, FACS,^8^ Lee Cranmer, MD, PhD,^9^ Eddy Hsueh, MD,^10^ Lisa Chen, PhD,^11^ Mark Shilkrut, MD, PhD,^11^ Howard L. Kaufman, MD, FACS^12^

^1^University of Utah, Huntsman Cancer Institute, Salt Lake City, UT; ^2^University of Texas M. D. Anderson Cancer Center, Houston, TX; ^3^Vanderbilt University Medical Center, Nashville, TN; ^4^University of Iowa Hospitals & Clinics, Iowa City, IA; ^5^University of North Carolina, Chapel Hill, NC; ^6^Emory University, Atlanta, GA ^7^Minnesota Oncology, Fridley, MN; ^8^Moffitt Cancer Center, Tampa, FL; ^9^University of Washington School of Medicine, Seattle, WA; ^10^Saint Louis University Cancer Center, St. Louis, MO; ^11^Amgen Inc., Thousand Oaks, CA; ^12^Rutgers Cancer Institute of New Jersey, Rutgers, NJ

Appendix 1 online

Baseline Demographics and Clinical Characteristics of Patients in the T-VEC Arm in OPTiM

| Characteristic | Patients With Durable Response | | | Intent to treat  N=295^a^ |
| --- | --- | --- | --- | --- |
|  | All  N=48 | Without PPR  N=25 | With PPR  N=23 |  |
|  |  |  |  |  |
| Median (range) age, years | 70 (38-91) | 74 (40-91) | 63 (38-86) | 63 (22-94) |
| Age — no. (%) |  |  |  |  |
| <65 years | 22 (46) | 9 (36) | 13 (57) | 152 (52) |
| ≥65 years | 26 (54) | 16 (64) | 10 (43) | 143 (48) |
| Sex — no. (%) |  |  |  |  |
| Men | 29 (60) | 16 (64) | 13 (57) | 173 (59) |
| Women | 19 (40) | 9 (36) | 10 (43) | 122 (41) |
| Disease stage — no. (%)^b,c^ | |  |  |  |
| IIIB | 10 (21) | 5 (20) | 5 (22) | 22 (8) |
| IIIC | 19 (40) | 9 (36) | 10 (44) | 66 (22) |
| IVM1a | 12 (25) | 7 (28) | 5 (22) | 75 (25) |
| IVM1b | 2 (4) | 1 (4) | 1 (4) | 64 (22) |
| IVM1c | 5 (10) | 3 (12) | 2 (9) | 67 (23) |
| LDH — no. (%)^b^ |  |  |  |  |
| ≤ULN | 44 (92) | 23 (92) | 21 (91) | 266 (90) |
| >ULN | 0 (0) | 0 (0) | 0 (0) | 15 (5) |
| Line of therapy — no. (%)^d^ |  |  |  |  |
| First line | 33 (69) | 18 (72) | 15 (65) | 138 (47) |
| Second or greater | 15 (31) | 7 (28) | 8 (35) | 157 (53) |
| ECOG performance status — no. (%)^b^ | | | | |
| 0 | 38 (79) | 18 (72) | 20 (87) | 209 (71) |
| 1 | 10 (21) | 7 (28) | 3 (13) | 82 (28) |
| Anatomic location of original disease — no. (%)^b,e^ | | |  |  |
| Scalp, face, neck | 21 (44) | 10 (40) | 11 (48) | 59 (20) |
| Trunk^f^ | 11 (23) | 6 (24) | 5 (22) | 72 (24) |
| Hand, arm | 2 (4) | 0 (0) | 2 (9) | 35 (12) |
| Leg, foot | 13 (27) | 7 (28) | 6 (26) | 107 (36) |
| Plantar/subungual | 1 (2) | 1 (4) | 0 (0) | 5 (2) |
| Other | 0 (0) | 0 (0) | 0 (0) | 21 (7) |
| *BRAF* status — no. (%) |  |  |  |  |
| Mutated | 5 (10) | 1 (4) | 4 (17) | 46 (16) |
| Wild-type | 5 (10) | 3 (12) | 2 (9) | 45 (15) |
| Unknown/ Missing | 38 (79) | 21 (84) | 17 (74) | 204 (69) |
| HSV-1 serostatus — no. (%)^b^ | |  |  |  |
| Negative | 13 (27) | 5 (20) | 8 (35) | 97 (33) |
| Positive | 31 (65) | 19 (76) | 12 (52) | 175 (59) |
|  |  |  |  |  |

PPR, Progression prior to response; ECOG, Eastern Cooperative Oncology Group; LDH, lactate dehydrogenase; ULN, upper limit of normal.

^a^ 4 patients did not receive T-VEC; ^b^ may have patients with unknown or missing data; ^c^ per CRF (case report form); ^d^ per IVRS (interactive voice response system); ^e^ the categories are not mutually exclusive; ^f^ chest, back, abdomen, pelvis.

Appendix 2 online

A. Distribution of All Baseline and New Measurable Lesions in the T-VEC Arm.


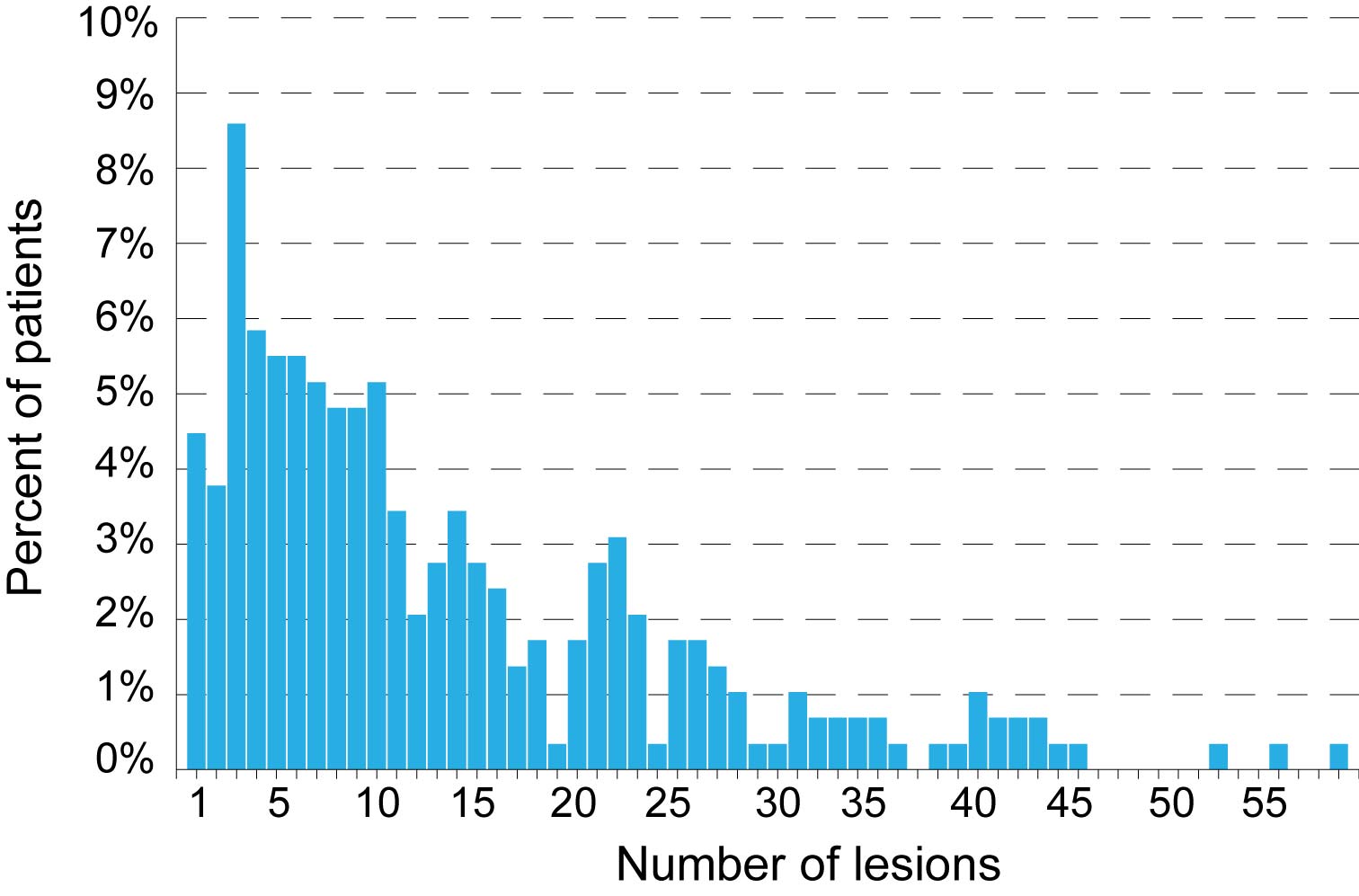


B. Distribution of Injected Lesions Per Patient in the T-VEC Arm.


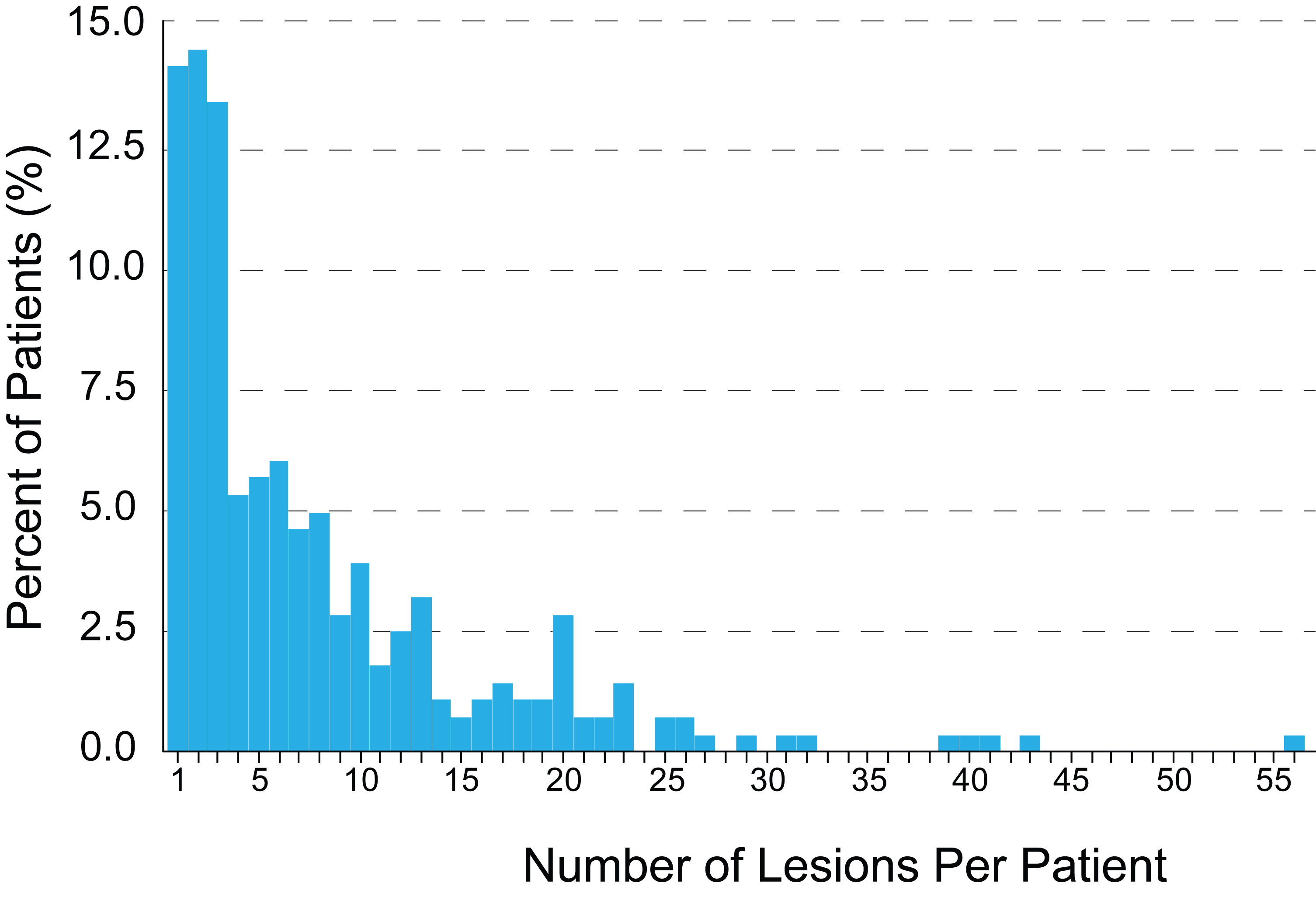


Appendix 3 online

Time-to-response of groups of responding lesions from baseline. For each plot, boxes represent lower and upper quartiles, the line represents the median, the diamond represents the mean, the whiskers represent boundaries of 1.5X below and above the lower and upper quartiles, respectively, and the open circles data points that are outside of the boundaries.


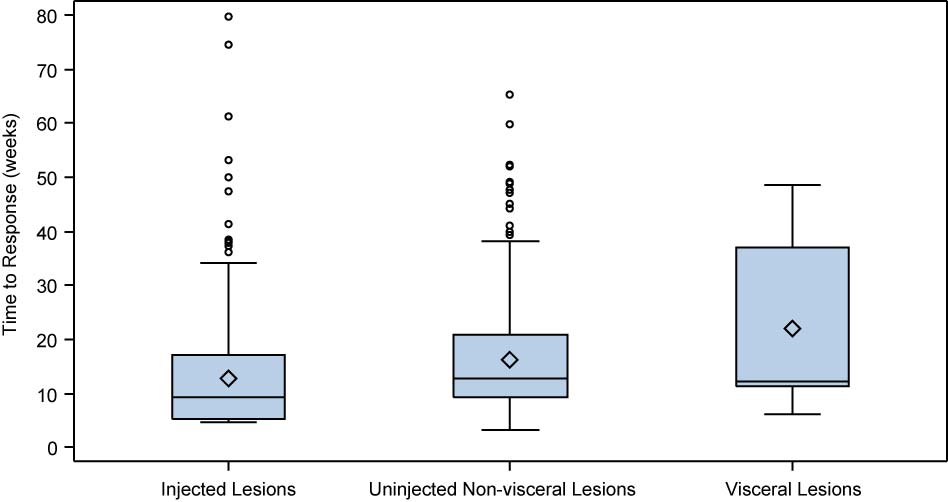


Appendix 4 online

Decrease in size of individual injected lesions, total tumor area of injected lesions in a patient-level analysis, and overall response in corresponding patients.

| Injected lesions^a^ (N=2116) | | Patients with injected lesions^b^ (N=277) | | | |
| --- | --- | --- | --- | --- | --- |
| Decrease in size of injected lesions | | Patients with a decrease in total tumor area of injected lesions | | Overall response^c^ | |
| ≥50% | 1361 (64%) | ≥50% | 102 (37%) | Overall response | 90 32%) |
| 100% | 995 (47%) | 100% | 45 (16%) | Complete response | 42 (15%) |

^a^ Lesions with at least 2 measurements recorded at two separate time points.

^b^ Patients with ≥1 lesion(s) with at least 2 measurements recorded at two separate time points.

^c^ Assessed by the investigators with modified WHO criteria (best overall response of all measurable tumors; overall response = complete + partial response). Partial response was defined as achieving a 50% or greater reduction in the sum of the products of the perpendicular diameters of all measurable tumors at the time of assessment as compared to the sum of the products of the perpendicular diameters of all measurable tumors at baseline.

Appendix 5 online

Response of individual uninjected non-visceral lesions by proximity to lesions injected with T-VEC. Lesions located in the same body site as injected lesions and lesions located in the different body site from injected lesions are shown. Vertical axis depicts change in individual tumor lesions size (products of the two largest perpendicular diameters) from baseline. Investigators assigned cutaneous, subcutaneous, and superficial nodal tumor lesions to 42-body sites grid defined by anatomical area (head; lower or upper arm; thigh; lower leg; foot dorsum or soles; upper, middle, and lower quadrants of the trunk) and side (front or back; left or right; top and side of head). Visceral lesions or deep lying nodal lesions (eg associated with visceral organs) were not assigned to the body site grid.

Among 981 uninjected, non-visceral evaluable lesions, 294 (30.0%) were located in the same body site as injected lesions, 306 (31.2%) were located in a different body site and 381 (38.8%) lesions had an unknown body site. Among 294 lesions located in the same body site as injected lesions, 159 (54.1%) decreased in size by ≥50% and 107 (36.4%) resolved completely. Among 306 uninjected, non-visceral lesions located in a different body site of injected lesions, 77 (25.2%) decreased in size by ≥50% and 39 (12.7%) resolved completely.


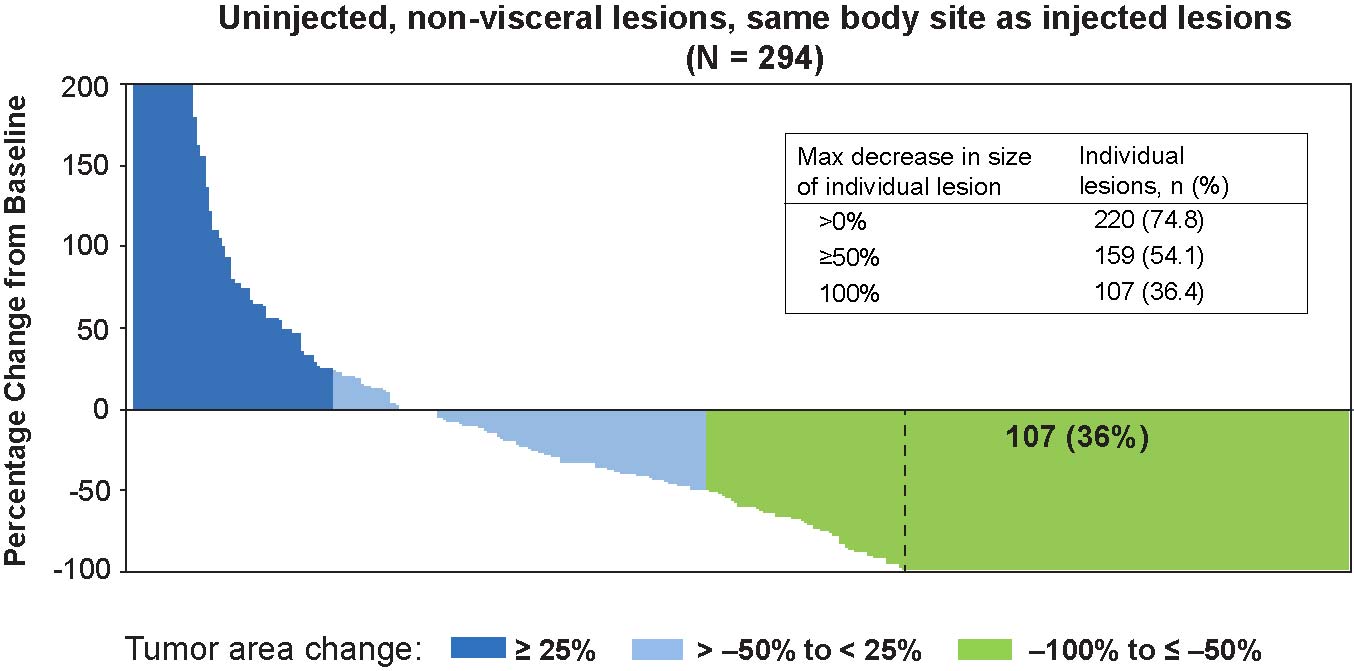


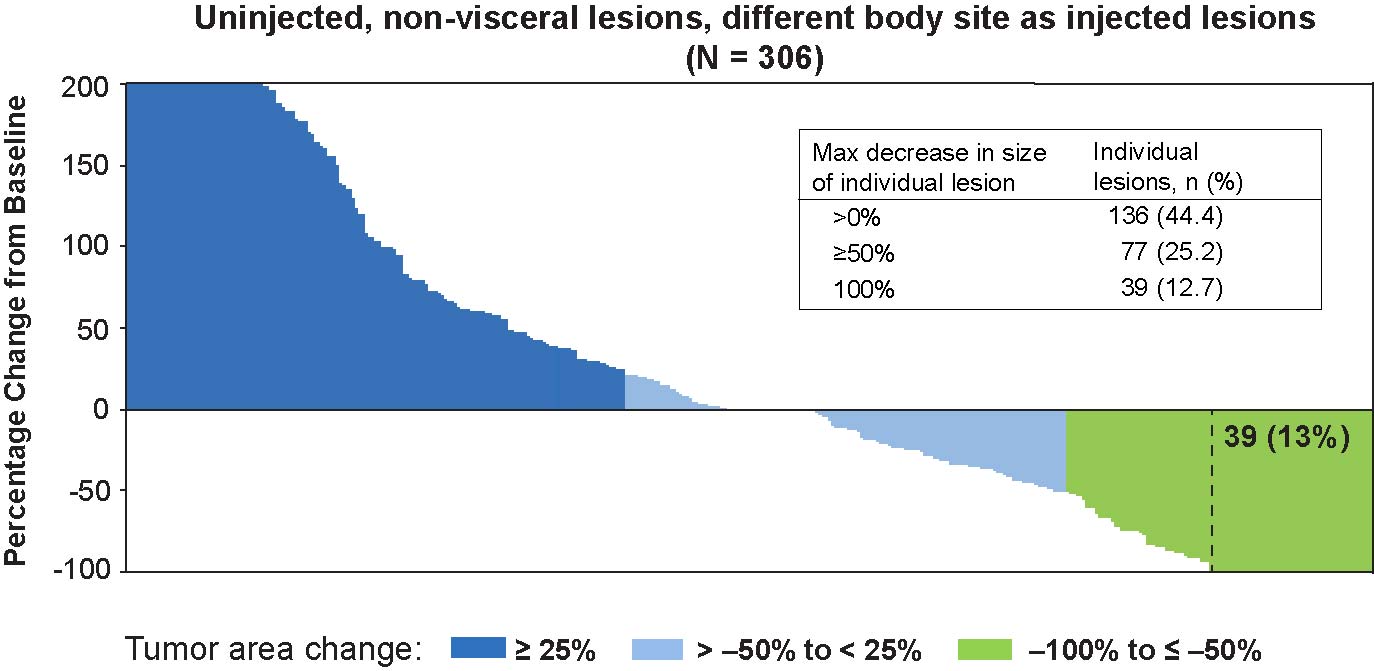
Appendix 6 online

Decrease in size of individual uninjected non-visceral lesions, total tumor area of injected lesions in a patient-level analysis, and overall response in corresponding patients.

| Uninjected non-visceral lesions^a^ (N=981) | | Patients with uninjected non-visceral lesions^b^ (N=177) | | | |
| --- | --- | --- | --- | --- | --- |
| Decrease in size of uninjected non-visceral lesions | | Patients with a decrease in total tumor area of uninjected non-visceral lesions | | Overall response^c^ | |
| ≥50% | 331 (34%) | ≥50% | 37 (21%) | Overall response | 31 (18%) |
| 100% | 212 (22%) | 100% | 24 (14%) | Complete response | 11 (6%) |

^a^ Lesions with at least 2 measurements recorded at two separate time points.

^b^ Patients with ≥1 lesion(s) with at least 2 measurements recorded at two separate time points.

^c^ Assessed by the investigators with modified WHO criteria (best overall response of all measurable tumors; overall response = complete + partial response). Partial response was defined as achieving a 50% or greater reduction in the sum of the products of the perpendicular diameters of all measurable tumors at the time of assessment as compared to the sum of the products of the perpendicular diameters of all measurable tumors at baseline.

Appendix 7 online

Decrease in size of individual visceral lesions, total tumor area of visceral lesions in a patient-level analysis, and overall response in corresponding patients.

| Visceral lesions^a^ (N=177) | | Patients with visceral lesions^b^ (N=79) | | | |
| --- | --- | --- | --- | --- | --- |
| Decrease in size of visceral lesions | | Patients with a decrease in total tumor area of visceral lesions | | Overall response^c^ | |
| ≥50% | 27 (15%) | ≥50% | 8 (10%) | Overall response | 11 (14%) |
| 100% | 16 (9%) | 100% | 5 (6%) | Complete response | 2 (3%) |

^a^ Lesions with at least 2 measurements recorded at two separate time points.

^b^ Patients with ≥1 lesion(s) with at least 2 measurements recorded at two separate time points.

^c^ Assessed by the investigators with modified WHO criteria (best overall response of all measurable tumors; overall response = complete + partial response). Partial response was defined as achieving a 50% or greater reduction in the sum of the products of the perpendicular diameters of all measurable tumors at the time of assessment as compared to the sum of the products of the perpendicular diameters of all measurable tumors at baseline.

Appendix 8 online

Kaplan-Meier plots of overall survival of patients with durable response (DR) and progression prior to response (PPR) versus patients with DR and without a PPR. Overall survival was calculated from the date of randomization to the date of death or last date when a patient was known to be alive. NE, not estimable.


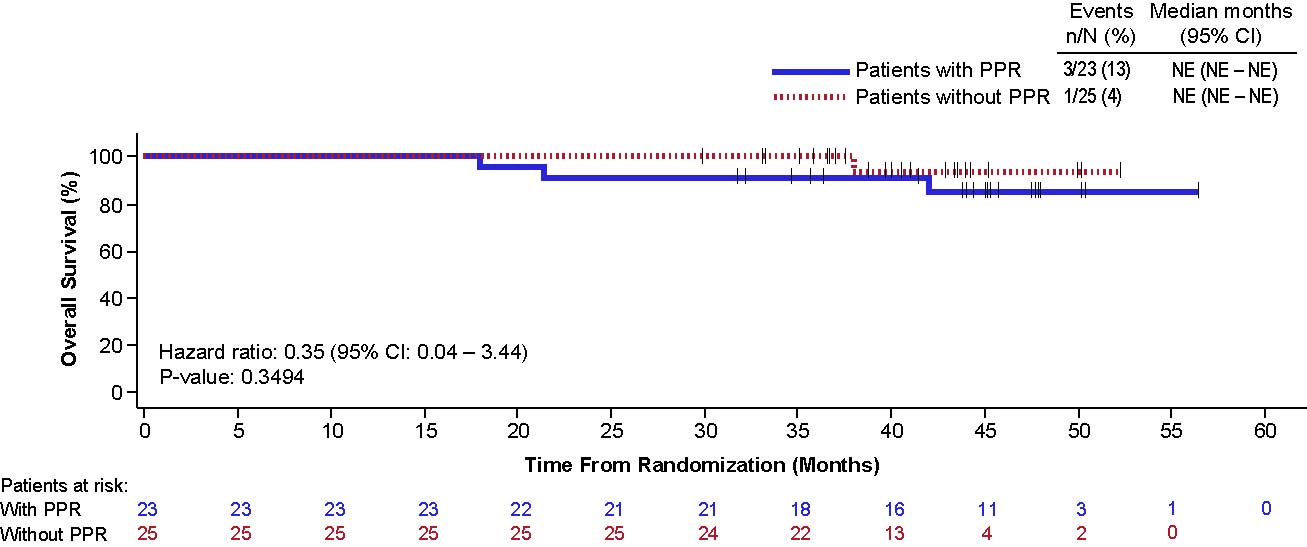


Appendix 9 online

Patient without progression prior to response (PPR) and durable response (DR) onset ≤6 months. The patient had newly diagnosed stage IVM1a melanoma with right axillary, retropectoral and infraclavicular (not shown) nodal metastases that were injected (axillary and retropectoral) with T-VEC under ultrasound guidance (arrows). Partial response was recorded on week 13 after the start of treatment and continued until the end of the study with DR duration of 59 weeks. All responses are per External Assessment Committee. Titles above each photography are weeks on study.


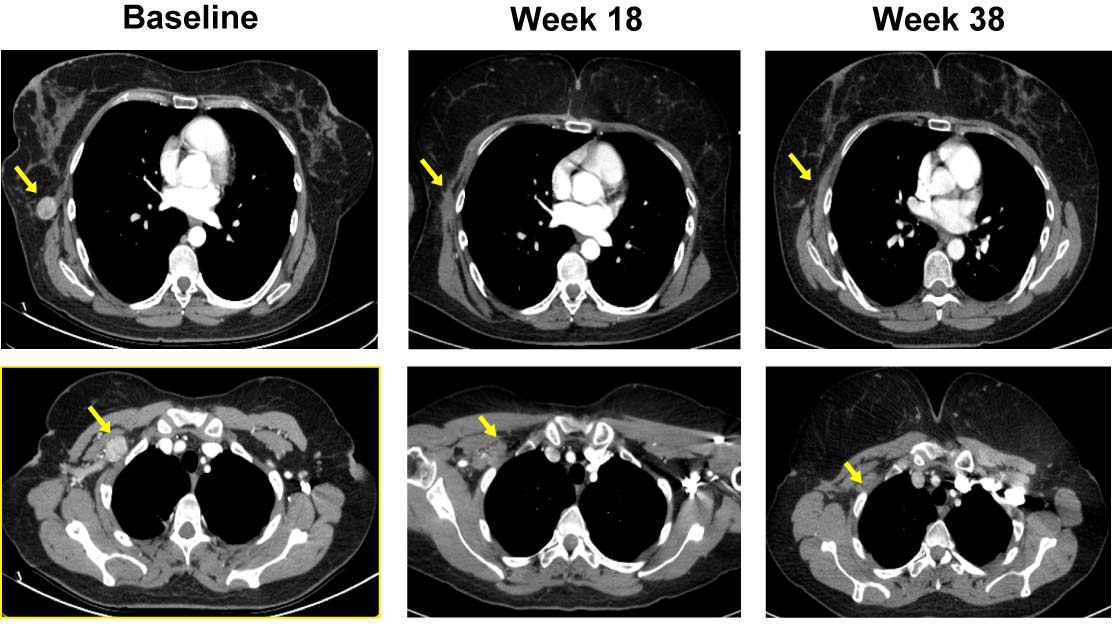
Appendix 10 online

Patient with progression prior to response (PPR) due to new lesions. The patient had recurrent stage IVM1a melanoma with multiple cutaneous metastases (15 measurable lesions and aggregates of lesions) and only three of the lesions at baseline (L1, L2 and L5) were injected with T-VEC. A new cutaneous lesion (yellow asterisk and arrow) appeared on week 17 after the start of treatment and was injected with T-VEC at 18 weeks. Partial response was recorded on week 37 after the start of treatment and all lesions, including the new lesion, resolved by week 50. The patient remained in complete response (confirmed by biopsy) until the end of the study with durable response duration of 41 weeks. All responses are per External Assessment Committee (EAC). Titles above each photography are weeks on study. Green arrows are marks of EAC.


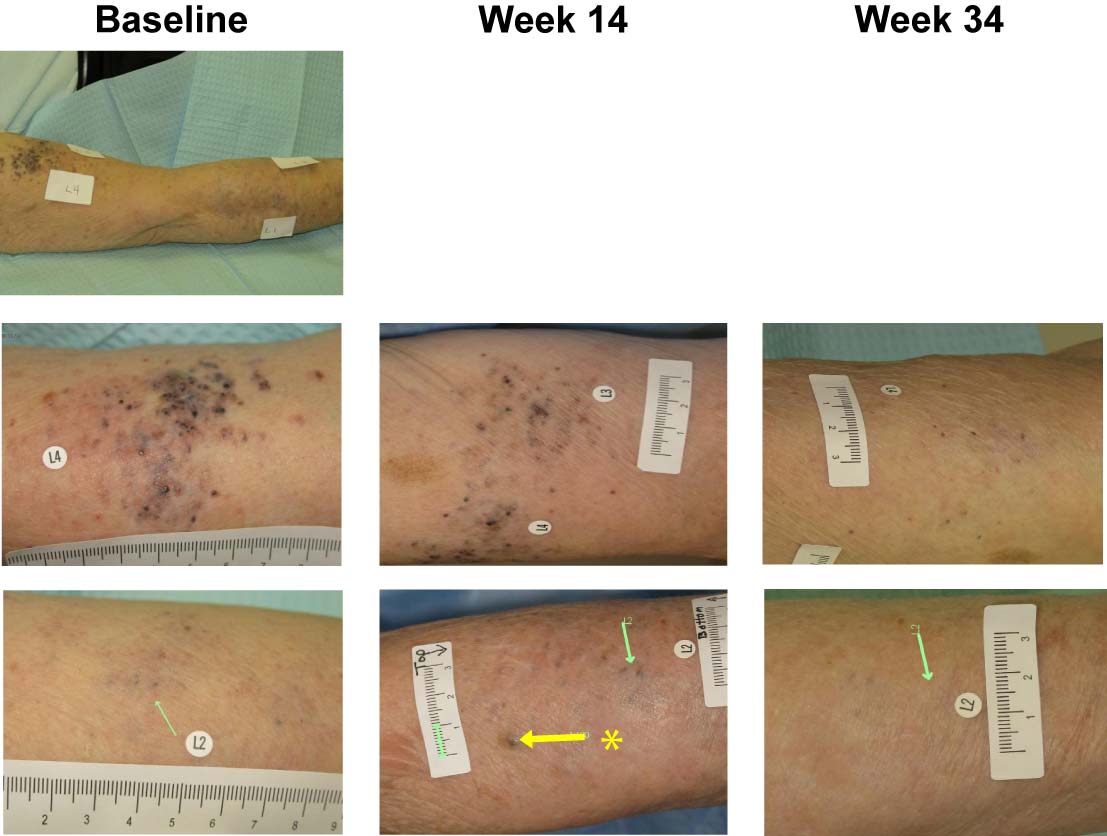

Supplement: Supplementary file 1 — Supplementary material 1 (DOCX 1260 kb) [file 10434_2016_5286_MOESM1_ESM.docx]
